# Protective Actions of Cannabidiol on Aging-Related Inflammation, Oxidative Stress and Apoptosis Alterations in Liver and Lung of Long Evans Rats

**DOI:** 10.3390/antiox12101837

**Published:** 2023-10-09

**Authors:** Lisa Rancan, Beatriz Linillos-Pradillo, Julia Centeno, Sergio D. Paredes, Elena Vara, Jesús A. F. Tresguerres

**Affiliations:** 1 Department of Biochemistry and Molecular Biology, School of Medicine, Complutense University of Madrid, 28040 Madrid, Spain; lisaranc@ucm.es (L.R.); beatlini@ucm.es (B.L.-P.); evaraami@ucm.es (E.V.); 2 Department of Physiology, School of Medicine, Complutense University of Madrid, 28040 Madrid, Spain; spared01@ucm.es

**Keywords:** CBD, aging, liver, lung

## Abstract

Background: Aging is characterised by the progressive accumulation of oxidative damage which leads to inflammation and apoptosis in cells. This affects all tissues in the body causing the deterioration of several organs. Previous studies observed that cannabidiol (CBD) could extend lifespan and health span by its antioxidant, anti-inflammatory and autophagy properties. However, research on the anti-aging effect of CBD is still in the beginning stages. This study aimed to investigate the role of cannabidiol (CBD) in the prevention of age-related alterations in liver and lung using a murine model. Methods: 15-month-old Long Evans rats were treated with 10 mg/kg b.w./day of CBD for 10 weeks and compared to animals of the same age as old control and 2-month-old animals as young control. Gene and/or protein expressions, by RT-qPCR and Western blotting, respectively, were assessed in terms of molecules related to oxidative stress (GST, GPx, GR and HO-1d), inflammation (NFκB, IL-1β and TNF-α) and apoptosis (BAX, Bcl-2, AIF, and CASP-1). In addition, MDA and MPO levels were measured by colorimetric assay. Results were analysed by ANOVA followed by Tukey–Kramer test, considering statistically significant a *p* < 0.05. Results: GST, GPx and GR expressions were significantly reduced (*p* < 0.01) in liver samples from old animals compared to young ones and CBD treatment was able to revert it. A significant increase was observed in old animals compared to young ones in relation to oxidative stress markers (MDA and HO-1d), proinflammatory molecules (NFκB, IL-1β and TNF-α), MPO levels and proapoptotic molecules (BAX, AIF and CASP-1), while no significant alterations were observed in the antiapoptotic molecules (Bcl-2). All these changes were more noticeable in the liver, while the lung seemed to be less affected. In almost all the measured parameters, CBD treatment was able to revert the alterations caused by age restoring the levels to those observed in the group of young animals. Conclusions: Chronic treatment with CBD in 15-month-old rats showed beneficial effects in lung and more significantly in liver by reducing the levels of inflammatory, oxidative and apoptotic mediators, and hence the cell damage associated with these three processes inherent to aging.

## 1. Introduction

Aging is a natural process influenced by various factors that can lead to a range of diseases due to cell dysregulation and organ dysfunction. A pressing issue is that, while the overall lifespan is increasing, the “health span”, or the period of life spent in good health, is not keeping pace, resulting in a global challenge of age-related diseases [1]. The study of aging biology has become a global pursuit in response to the demands of our time. Presently, there are several identified hallmarks related to the aging process, including genomic instability, stem cell exhaustion and mitochondrial dysfunction [2], which lead to a decrease in energy production in the form of ATP and an increase in the generation of reactive oxygen species (ROS) [3]. This, together with a decrease in the cell’s antioxidant defences, creates a state of oxidative stress, which compromises the cell’s viability in several ways.

In a young, healthy cell, free radicals inherent to the cell’s metabolism are scavenged by cellular enzymatic antioxidants, preventing oxidative damage. Amongst these enzymes, glutathione peroxidase (GPx) (BRENDA:EC1.11.1.9) is also involved in antioxidant defence, as it oxidises glutathione to reduce hydrogen peroxide and organic hydroperoxides. The oxidated glutathione (GSSG) levels are restored by the action of the glutathione reductase (GR) (BRENDA:EC1.8.1.7), an antioxidant enzyme which is essential to maintain the GSH/GSSG. Another important antioxidant enzyme is Glutathione S-transferase (GST) (BRENDA:EC2.5.1.18), which conjugates glutathione to electrophilic substrates such as lipid peroxides, increasing their solubility in water and facilitating their elimination. In this way, GST protects the cell from oxidative damage. Another important enzyme in oxidative stress conditions is heme-oxygenase (HO), which catalyses the degradation of heme. HO has two isoforms, including HO-1 (BRENDA:EC1.14.14.18), which is especially interesting as its expression is induced under oxidative stress so that it carries out its antioxidant and anti-inflammatory action [4]. There are also non-enzymatic antioxidants, such as vitamin E and vitamin C, which help to prevent the accumulation of free radicals. Altogether, the cell’s antioxidant defence systems maintain cellular redox balance, avoiding the damage of DNA, lipids and proteins caused by the production of free radicals.

As an organism ages, antioxidant systems decline, increasing the risk of free radicals acting on their target molecules. Moreover, ROS production increases due to mitochondrial dysfunction, due to mutations accumulated with time in mtDNA [5]. When free radicals exceed the cell’s antioxidant capacity, many molecules in the cell are altered. Proteins are highly sensitive to oxidative damage, resulting in changes in their structure and function [6]. Nucleic acids also undergo modifications under oxidative stress, for example, damaged DNA has been shown to increase levels of 8-hydroxy-2′-deoxyguanosine (8-OH-dG), which serves as a biomarker for oxidative damage [7]. Lipids are also damaged by oxidation through a process known as lipid peroxidation. This is a chemical process that occurs when free radicals react with polyunsaturated fatty acids (PUFAs) found in cell membranes. This reaction results in the formation of lipid peroxides, such as malonyl aldehyde (MDA), which are highly reactive and can further react with other molecules, leading to damage and dysfunction in cells and tissues. Oxidative stress has been proven to trigger several signalling pathways leading to an increase in inflammation as well as in apoptosis in several pathological situations, such as diabetes mellitus [8]. Aging is thus associated with an increase in oxidative stress, inflammation and apoptosis, which altogether lead to the dysfunction and degeneration of cells, tissues and organs [9].

As mentioned, the disturbance of the oxidative–antioxidant balance may play a significant role in the pathogenesis of inflammation. An example is the role of free radicals, such as H_2_O_2_, as second messengers in regulating the activation of transcription factors including nuclear factor kappa B (NFκB) [10]. NFκB is a group of proteins involved in the immune response to infection and inflammation, which includes NFκB-p65, NFκB-p50 and NFκB-p52. In the presence of inflammation or oxidative stress, signalling pathways lead to the degradation of these proteins and NFκB translocates to the nucleus and activates several genes, including IL-1β (interleukin-1 beta) and TNF-α (tumour necrosis factor alpha), cytokines involved in the immune response to infection, injury and inflammation, which can also activate NFκB themselves [11]. TNF-α and IL-1β can also favour the increase in ROS by activating NOX (NADPH oxidase) [12]. NFκBp52, on the other hand, can activate the transcription of genes involved in inflammation and in cellular senescence [13].

Although inflammation is an essential protective response to injury, chronic inflammation can cause irreversible tissue damage. Together with oxidative stress, it is considered a major cause of age-related diseases and cancer [14]. Although this damage affects all tissues, liver is particularly sensitive as it is involved in local and systemic inflammatory response, for which it expresses a higher number of receptors involved in these signalling cascades that make it a more sensitive target [15]. On the other hand, human lungs, the organ with the largest surface area in the body, are also exposed to inflammatory diseases that have been attributed to age-related changes in innate and adaptive immune responses in these organs [16]. Lungs represent a unique interface with the outside environment and, although drivers of lung aging remain elusive, it seems that oxidative stress plays an important role in the pathogenesis of age-related pulmonary inflammatory diseases including pulmonary hypertension, fibrotic lung disease, and COPD [17].

As a consequence of oxidative stress, several apoptosis signalling pathways can be activated. For example, DNA damage leads to a change in ratio between the proteins of the Bcl-2 family, some of which are proapoptotic (like Bcl-2-associated X protein (BAX) and Bcl-2 antagonist/killer (BAK)) and some are antiapoptotic ones (like B-cell lymphoma 2 (Bcl-2) and B-cell lymphoma—extra-large (Bcl-XL)). When this happens, BAX and BAK form pores in the mitochondrial outer membrane, causing its permeabilisation (MOMP), which leads to the release of cytochrome c, ATP and the apoptosis inducing factor (AIF) among others. AIF is a protein that, when released from the mitochondria, can reach the nucleus and cause DNA fragmentation, triggering apoptosis through a caspase-independent pathway. A correlation between age and the induction of this protein has been found in human skeletal muscle [18]. Oxidative stress can also trigger apoptosis due to the accumulation of ROS in the mitochondria, which also favours the formation of pores in its membrane and leads to the release of cytochrome c into the cytoplasm [19]. Moreover, TNF-α, one of the cytokines expressed due to the inflammatory state of the cell, can activate the extrinsic pathway of apoptosis by binding to death receptors and activating caspase 8 [20]. In general, inflammation, apoptosis and oxidative stress share several signalling pathways that cause a harmful cycle of damage to cells, which progressively lose viability and thus lead to the aging of tissues and organs.

Hence, promising strategies for counteracting aging include reducing age-related inflammation and oxidative stress, enhancing autophagy and regulating the gut microbiome [21]. Various anti-aging compounds have been investigated and, among them, natural medicinal plant compounds hold a significant position [22].

Cannabidiol (CBD) (DrugBank accession number: DB09061) is a molecule found in Indian hemp, which is legal in most European countries because, unlike other hemp constituents such as delta-9-tetrahydrocannabinol (THC), it does not have psychotropic effects. The increasing number of benefits found for this molecule makes it an interesting target for medical research. The antioxidant and anti-inflammatory effects of CBD make it an interesting target for “anti-aging” treatment. In fact, CBD was able to reduce oxidative stress, inflammation and cell death in a mouse model of type I diabetic cardiomyopathy [23]. Moreover, CBD has been studied as a potential therapeutic strategy for the treatment of Parkinson’s disease, an age-related neurodegenerative disease, in which cellular homeostasis is compromised, leading to an increase in oxidative stress and inflammation [24].

CBD can act as an antioxidant at several levels, either through interaction with its receptors or in a receptor-independent way [12]. CBD can have direct antioxidant action through the scavenging of ROS such as H2O2. It can also donate electrons to transform ROS into more inert molecules which are less harmful for the cell and easier to eliminate. CBD has hydroxyl groups which increase its highest occupied molecular orbital, and thus its capacity to act as a powerful antioxidant [25]. Moreover, CBD can chelate transition metals necessary for Fenton reactions, which are behind the non-enzymatic production of ROS [26].

The activation of these pathways is closely related to CBD’s anti-inflammatory action. For example, the action of Nrf2, favoured by CBD, also leads to a decrease in IL-1β in the macrophages and TNF-α in Th1 lymphocytes [27]. Moreover, CBD can inhibit TNF-α and NFκB expression in cardiac tissue following doxorubicin cardiotoxicity [12]. Apart from this, a structural analogue of CBD was demonstrated to reduce the production of TNF-α through the inactivation of NFκB [28].

Apart from this, CBD has also been shown to reduce inflammation associated with the activity of the inflammasome, as evidence shows it can reduce the expression of NLRP3 inflammasome-related genes, leading to a decrease in IL-1β levels [29]. CBD also led to a decrease in the expression of caspase 3 after doxorubicin cardiotoxicity [12], which suggests it can also act to reduce the high apoptotic rate associated with aging. All this evidence regarding CBD’s antioxidative, anti-inflammatory and anti-apoptotic action suggests that it can protect the cell from the molecular damage associated with the aging process [30].

Under the premise that aging increases inflammation, oxidative stress, and apoptosis, causing cellular damage, and that CBD has shown antioxidant and anti-inflammatory properties in different physiological and pathological contexts, the aim of this work was to analyse the effect of chronic CBD treatment in liver and lung in a Long Evans rat model of aging.

## 2. Materials and Methods

### 2.1. Animal Model and Treatment

For this study, 40 Long Evans rats (Janvier Labs, Le Genest Saint Isle, France) were used. Two-month-old rats served as young controls, and 15-month-old rats were used as old rats to study aging alterations. During the study, the animals were housed in the Animal Facility of the Complutense University of Madrid located at the School of Medicine (Registration No: ES-28079-0000086). The project complied with the provisions of Royal Decree 53/1 February 2023, which establishes the basic rules applicable to the protection of animals used for experimentation and other scientific purposes.

Regarding the diet, all young rats (*n* = 11) and untreated old rats (*n* = 15) received standard food, while the treated old rats (*n* = 14) were fed with CBD-enriched chow for two months. For its preparation, pure CBD extract (Phexia, Madrid, Spain) was used to create a stock solution of CBD, which was then mixed with standard chow to obtain a CBD concentration of 200 mg/100 g. The dosage of the treatment was 10 mg/kg of body weight (b.w.) per day, and the rats’ weight was measured weekly, with the daily food intake volume was recorded. At the end of the treatment period, the animals were euthanised by decapitation with a guillotine. For this study, the tissues of interest (liver and lung) were frozen in liquid nitrogen and stored at −80 °C until analysis.

### 2.2. mRNA Isolation and RT-PCR Quantification

The mRNA expression of BAX, NFκB-p65, NFκB-p52, NFκB-p50, GST, GPx, IL-1β, TNF-α, AIF, HO-1d, GR, and CASP-1 was determined by measuring the relative levels of their messenger RNAs using the real-time quantitative PCR (RT-qPCR) technique. RNA was isolated from liver and lung biopsies according to the methods described by Chomczynski [31] using the TRI Reagent Kit (Molecular Research Center, Inc., Cincinnati, OH, USA) following the manufacturer’s protocol. The purity of the RNA was estimated by 1% agarose gel electrophoresis, and RNA concentrations in the ratio 260/280 were determined by spectrophotometry BioDrop (Fisher scientific, Waltham, MA, USA). Reverse transcription of 2 mg of RNA for cDNA synthesis was performed using the StaRT Reverse Transcription Kit (AnyGenes, Paris, France). qRT-PCR was performed using a 7500 Fast Real Time PCR System thermal cycler (Applied Biosystems, Cambridge, MA, USA) with the TB Green Ex Taq (Tli RNase H Plus) (Takara Bio Inc., Kusatsu, Shiga, Japan) and 300 nM concentrations of specific primers (Table 1). The qPCR amplification cycles were a 95 °C 10 min cycle, followed by 45 cycles at 95 °C for 10 s and at 60 °C for 30 s and finally melting curve analysis, following the recommendations of the manufacturer (95 °C for 10 s, 65 °C for 30 s, and 95 °C for 30 s). Amplification of 18S mRNA was used as a loading control for each sample. The gene expression level was analysed in triplicate for each sample. Relative changes in mRNA expression were calculated using the 2^−∆∆CT^ method [32].

### 2.3. Protein Extraction and Western Blotting Analysis

The expression of TNF-α, NFκB p65, NFκB p52, AIF and Bcl-2 proteins was measured using the Western blotting technique. In summary, liver and lung samples were homogenised using a modified RIPA lysis buffer (consisting of PBS, Igepal, sodium deoxycholate, 10% SDS, PMSF, 0.5 M EDTA and 100 mM EGTA), supplemented with protease inhibitor cocktail, PMSF (1 mM), sodium orthovanadate (2 mM), and sodium pyrophosphate (20 mM) (Sigma-Aldrich, Madrid, Spain). After sonication and boiling at 100 °C for 10 min in a 1:1 ratio with gel-loading buffer (containing 100 mmol/L TrisHCl at pH 6.8, 4% SDS, 20% glycerol, 0.1% bromophenol blue, and 200 mmol/L dithiothreitol), total protein equivalents (25 μg) for each sample were separated by SDS-PAGE using 10% Mini-PROTEAN TGX Precast acrylamide gels (Bio-Rad Laboratories, Hercules, CA, USA). After electrophoresis, Stain Free technology was activated using the BioRad^®^ Chemi-Doc MP Imaging System (Bio-Rad Laboratories, Hercules, CA, USA) and were transferred onto a PVDF membrane using the Trans-Blot^®^ Turbo™ Transfer System (Bio-Rad Laboratories, Hercules, CA, USA). Following transfer, the membrane was immediately placed in a blocking buffer containing 5% non-fat milk in 20 mM Tris at pH 7.5, 150 mM NaCl, and 0.01% Tween-20, and allowed to block at 37 °C for 1 h. Subsequently, the membrane was incubated with a rabbit polyclonal antibody (Table 2) for 12 h at 4 °C, followed by incubation with a goat anti-rabbit IgG secondary antibody (Santa Cruz Biotechnology, Santa Cruz, CA, USA) (1:7000). To detect proteins, the Clarity Western ECL Substrate assay kit (Bio-Rad Laboratories, Hercules, CA, USA) and/or the ECL Plus (Amersham Life Science Inc., Buckinghamshire, UK) were utilised for chemiluminescence with the BioRad ChemiDoc MP Imaging System, allowing the determination of the relative optical densities. Pre-stained protein markers were employed to determine molecular weights.

The intensity of the bands present in each lane was quantified using BioRad Image Lab software v6.1 (Bio-Rad Laboratories, Hercules, CA, USA) and the normalisation of results was performed using the total amount of protein loaded in each well (based on the Stain-Free technology from Bio-Rad Laboratories, Hercules, CA, USA).

### 2.4. Thiobarbituric Acid Reactive Substances (TBARS) Assay

Lipid peroxidation was also evaluated using a commercial kit (BioVision, Mountain View, CA, USA) that measures the reaction of malondialdehyde (MDA) with thiobarbituric acid (TBA) and the formation of MDA-TBA adducts. The samples were resuspended in a lysis buffer containing the antioxidant butylated hydroxytoluene (BHT) (0.1 mM) to prevent the formation of MDA during sample preparation or the heating phase. Then, they were centrifuged at 3200× *g* for 30 min. Furthermore, 200 μL of supernatant from each sample was added to 600 μL of TBA and incubated at 95 °C for 60 min. The samples were cooled on ice for 10 min, and 300 μL of n-butanol (Sigma-Aldrich, Madrid, Spain) was added to create an organic phase where MDA molecules would form. The samples were centrifuged, and 200 μL of the upper organic phase was collected and dispensed into a 96-well microplate for spectrophotometric measurement at 532 nm. The results were expressed as nmol TBARS/mg protein.

### 2.5. Histological Staining

Liver tissues were washed in 0.9% cold saline and fixed in a 10% formalin buffer solution for the histopathological assessment for 24 h. After fixation, samples were processed for embedding in paraffin. Serial sections (5 µm) were prepared using a rotary microtome Leica RM2125 RTS (Leica Biosystems, Wetzlar, Germany) for haematoxylin and eosin staining (H&E). The sections were stained with 0.1% haematoxylin (Ciba, Basel, Switzerland) for 5 min. Then, slides were washed with tap water for 15 min and then a quick wash with hydrochloric alcohol (0.5% HCl in absolute ethanol) to remove excess staining on the sample (differentiation). The acid was neutralised by immersing the sections in tap water for 5 min and a final wash with distilled water. They were immersed in 0.1% eosin (Ciba, Basel, Switzerland) for 5 min. After washing with distilled water, tissue sections were dehydrated using ascending ethanol passages and finishing in xylol for 30 s. Tissue sections were cover slipped. Images were captured with Leica Microscope (Leica Biosystems, Wetzlar, Germany).

### 2.6. Myeloperoxidase Cuantification

Levels of myeloperoxidase (MPO) were measured in liver samples using specific ELISA kit (MyBiosource, San Diego, CA, USA. Cat. N°: MBS704859) by following the manufacturer’s instructions. Briefly, 50 mg of liver biopsies were homogenised in 500 μL of PBS. Then, standards and samples were pipetted into pre-coated wells, and MPO present in the samples was bound by the immobilised antibody. After removing any unbound substances, a biotin-conjugated antibody specific for the investigated protein was added to the well. After washing, avidin-conjugated horseradish peroxidase was added to the wells. Following a wash to remove any unbound avidin–enzyme reagent, a substrate solution was added to the wells. After 10 min, the colour development was stopped and the intensity of the colour was measured at 450 nm. The intra-assay CV was <8% and inter-assay CV < 10%. The results were expressed as ng/mL.

### 2.7. Statistical Analysis

The differences between the obtained values, presented as mean ± SD, were evaluated using one-way analysis of variance (ANOVA) followed by Tukey–Kramer multiple comparisons test to compare all pairs of means after conducting a normality test. A confidence level higher than 95% (*p* < 0.05) was considered statistically significant. Prism v8 software (GraphPad Software, Inc., San Diego, CA, USA) was used for the statistical calculations.

## 3. Results

### 3.1. Expression of Antioxidant Enzymes and Oxidative Stress Markers

**Figure 1:**
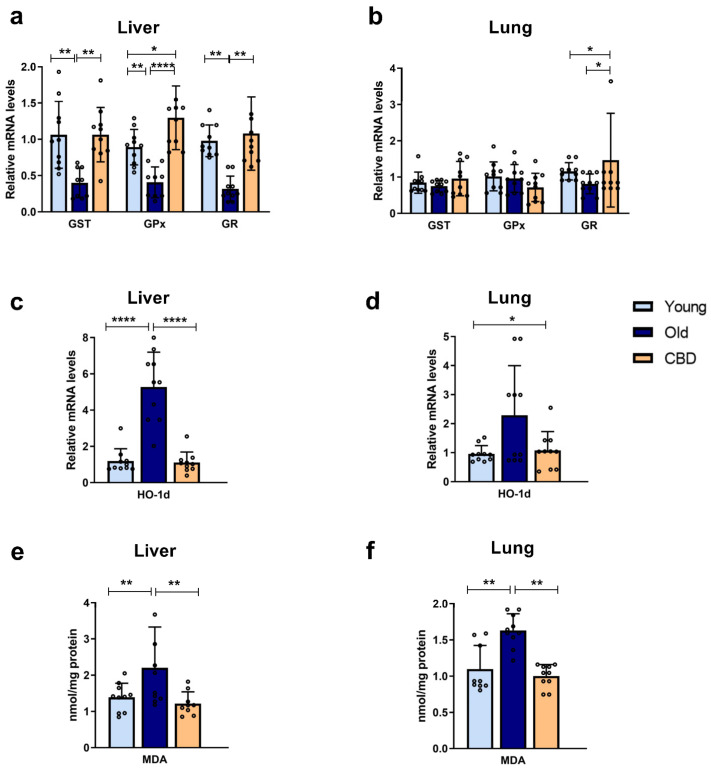
Expression of antioxidant enzymes and oxidative stress markers. (**a**) mRNA expression of GPx, GST and GR in liver samples; (**b**) mRNA expression of GPx, GST and GR in lung samples; (**c**) mRNA expression of HO-1d in liver samples; (**d**) mRNA expression of HO-1d in lung samples; (**e**) Concentration of MDA in nmol/mg protein in liver samples; (**f**) Concentration of MDA in nmol/mg protein in lung samples. Data represent mean ± SD. Individual data points are also shown. Light blue bars represent young group, dark blue bars represent old group and yellow bars represent the group of old animals treated with CBD. *n* = 10 rats per experimental group. * *p* < 0.05; ** *p* < 0.01; **** *p* < 0.0001.

At the hepatic level, a significant decrease (*p* < 0.01) in the mRNA expression of antioxidant enzymes GST, GPx and GR was observed in the group of old rats compared to the young rats. Treatment with CBD was able to reverse this effect, with values equal to or even significantly higher than those of the young group (Figure 1a). At the pulmonary level, the aging-related alterations were not observed. In addition, CBD treatment showed no significant differences in GST and GPx expressions but a significant increase in GR was observed in the group of old rats treated with CBD compared to the other groups (Figure 1b).

Regarding the HO-1d mRNA expression, a highly significant increase (*p* < 0.0001) was observed in the liver samples of the old group compared to both the young and the treated animals (Figure 1c), while at the pulmonary level, this rise was not significant. A significant rise was observed in the CBD group compared to the young group (*p* < 0.05), although this was quantitatively lower, but not significantly, than the levels seen in the old animals (Figure 1d).

A significant increase in lipid peroxidation was observed in the group of old rats with respect to the group of young rats (*p* < 0.001), both in the liver and lung samples. Also, in both cases, a significant decrease (*p* < 0.001) was observed in the CBD group, whose levels were similar to those of the young group (Figure 1e,f).

Taken together, these results suggest that aging provoked an increase in oxidative stress associated with a decrease in antioxidant capacity. Furthermore, CBD was able to counteract these elevations.

### 3.2. Inflammation Markers

**Figure 2:**
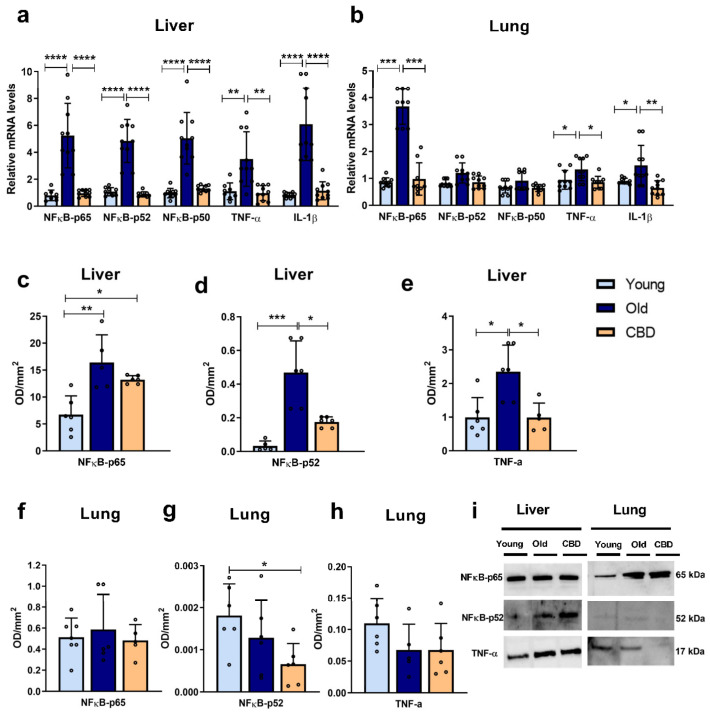
Expression of inflammation markers. (**a**) mRNA expression of NFκB in its different units (p65, p52 and p50), TNF-α and IL-1β in liver samples; (**b**) mRNA expression of NFκB in its different units (p65, p52 and p50), TNF-α and IL-1β in lung samples; (**c**) Protein expression of NFκB-p65 in liver samples; (**d**) Protein expression of NFκB-p52 in liver samples; (**e**) Protein expression of TNF-α in liver samples; (**f**) Protein expression of NFκB-p65 in lung samples; (**g**) Protein expression of NFκB-p52 in lung samples; (**h**) Protein expression of TNF-α in lung samples; (**i**) Representative images of the Western blotting results of the different proteins studied. Data represent the mean ± SD. Individual data points are also shown. Light blue bars represent the young group, dark blue bars represent the old group and the yellow bar represent the group of old animals treated with CBD. For the mRNA expression, *n* = 10 rats per experimental group; for protein expression, *n* = 6 rats per experimental group. * *p* < 0.05; ** *p* < 0.01; *** *p* < 0.001; **** *p* < 0.0001.

In liver samples, the mRNA expression of inflammatory markers (NFκB-p65, NFκB-p52, NFκB-p50, TNF-α and IL-1β) was highly and significantly increased (*p* < 0.001 and *p* < 0.0001) in the old animal group compared to both the young and treated animals (Figure 2a). In contrast, in lung samples, significant differences were only observed in the expressions of NFκB-p65, TNF-α and IL-1β. In all three markers, a significant increase (*p* < 0.0001, *p* < 0.05 and *p* < 0.01, respectively) was observed in the old group of animals compared to both the young and treated ones (Figure 2b). These results were mainly confirmed when protein expressions were analysed in the liver. Thus, a significant rise caused by aging was observed in all the measured markers (NFκB-p65, NFκB-p52 and TNF-α) (Figure 2c–e), but not in the lung (Figure 2f–h). The treatment with CBD was able to reduce these aforementioned alterations in liver, restoring the markers to similar levels seen in the young group, with the exception of the protein expression of NFκB-p65, which was higher in the treated group compared to the young one (*p* < 0.05) (Figure 2c). As for the lung, only a significant decrease was observed in the CBD-treated group in comparison with the young animals (*p* < 0.05) (Figure 2g).

Taken together, these results suggest that aging caused an increase in some proinflammatory mediators and that this increase affected the liver more than the lung. Moreover, CBD treatment seemed to reverse these effects without altering those markers that had not undergone any aging-associated changes.

### 3.3. Apoptosis Markers

**Figure 3:**
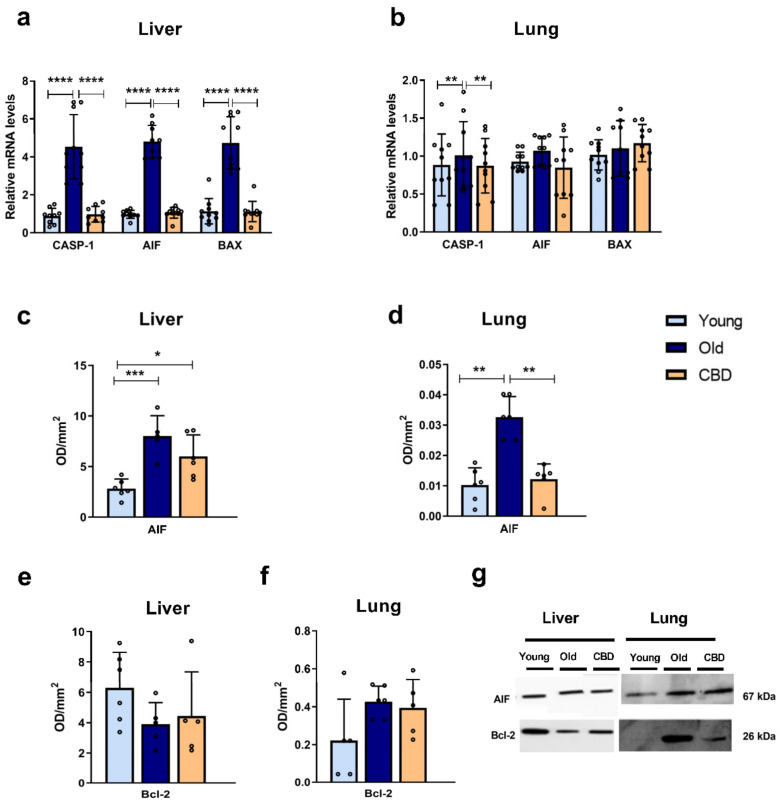
Expression of apoptosis markers. (**a**) mRNA expression of CASP-1, AIF and BAX in liver samples; (**b**) mRNA expression of CASP-1, AIF and BAX in lung samples; (**c**) Protein expression of AIF in liver samples; (**d**) Protein expression of AIF in lung samples; (**e**) Protein expression of Bcl-2 in liver samples; (**f**) Protein expression of Bcl-2 in lung samples; (**g**) Representative images of the Western blotting results of the different proteins studied. Data represent the mean ± SD. Individual data points are also shown. Light blue bars represent the young group, dark blue bars represent the old group and the yellow bar represents the group of old animals treated with CBD. For the mRNA expression, *n* = 10 rats per experimental group; for protein expression, *n* = 6 rats per experimental group. * *p* < 0.05; ** *p* < 0.01; *** *p* < 0.001; **** *p* < 0.0001.

At the hepatic level, the mRNA expression of CASP-1, AIF and BAX was significantly higher in the old group compared to both the young group and the CBD-treated group (*p* < 0.0001) (Figure 3a). In contrast, at the pulmonary level, this rise was only observed in the CASP-1 mRNA expression (*p* < 0.05), while no significant differences were observed in the other markers (Figure 3b).

Regarding the protein expression, a significant increase was observed in AIF in the old group compared to the young one in both liver (*p* < 0.001) and lung (*p* < 0.01) samples (Figure 3c,d). CBD treatment was able to significantly reduce this rise in the lung (*p* < 0.01) (Figure 3d) while only a tendency was observed in the liver samples, where the CBD-treated group still showed significantly higher AIF protein expression than the young group (*p* < 0.05) (Figure 3c).

No significant changes were observed in liver or lung samples when the antiapoptotic protein Bcl-2 was analysed (Figure 3e,f).

Taken together, these results suggest that aging was associated with an increase in the studied pro-apoptotic mediators and again that this increase affected the liver more than the lung. Moreover, CBD treatment was able to prevent these effects with no alteration of markers that had not undergone any aging-associated changes.

### 3.4. Histopathology and MPO Levels

**Figure 4:**
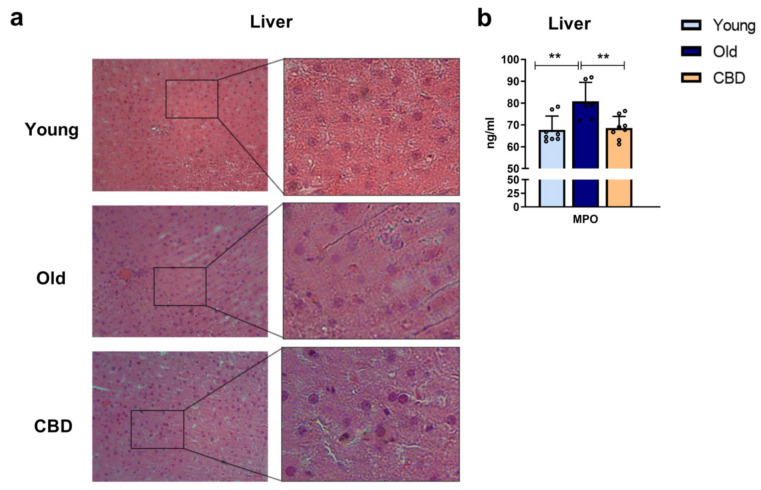
(**a**): Histology of liver sections. Representative images of livers from young, old and CBD-treated animals stained with H&E. Images indicate 200 µm (20×) and the magnified image of the characteristic tissue section (40×). (**b**): Concentration of MPO in ng/mL in liver samples. Data represent mean ± SD. Individual data points are also shown. Light blue bar represents young group, dark blue bar represents old group and yellow bar represents the group of old animals treated with CBD. *n* = 8 rats per experimental group. ** *p* < 0.01.

Haematoxylin and eosin staining was used to analyse the effect of CBD on liver morphology and the MPO concentration was measured as a marker of activated neutrophils. While no significant alterations were observed in the sections of young and old group animals as well as in the CBD-treated group when H&E staining was performed (Figure 4a), MPO levels showed significantly higher concentrations in old animals compared to the young group. Also, a significant decrease was observed when old animals were treated with CBD (Figure 4b).

## 4. Discussion

The aging process in an individual is characterised by the progressive deterioration of organs and the consequent loss of their function. Although the rate at which this deterioration occurs varies between each individual, the attempt to slow down this inevitable process is a common and growing interest among the human population [1]. With this approach, medicine has been searching for the molecular causes that lead to cellular aging and, therefore, organismal aging. Although part of the variability in the rate of aging is genetic, it is a multifactorial process that is also influenced by lifestyle and other exogenous factors [33]. This allows for the exploration of molecules that can reduce the damages associated with aging and, thus, slow down the process, which many refer to as “anti-aging therapy” [21]. Among the nine pillars of aging [2], mitochondrial dysfunction is characterised by increased oxidative stress, which results in the activation of inflammation and apoptosis pathways, leading to tissue damage and degeneration. This is particularly interesting in the context of “anti-aging therapy” because it suggests the potential use of antioxidants and anti-inflammatory agents to reduce age-related pathology. CBD is the second most abundant active ingredient in cannabis and has great therapeutic potential. It not only lacks psychotropic effects but has also been shown to have antidepressant, sleep-inducing, antioxidant, and anti-inflammatory effects [25,26]. This has led to its increasing legal use for medicinal purposes in many countries, making it a molecule with potential protective effects against age-related damage. Therefore, this study examined the effects of oral CBD administration on oxidative stress, inflammation, and apoptosis processes in liver and lung tissues in a murine aging model.

In the livers of old Long Evans rats, a decrease in the expression of antioxidant enzymes GR, GPx, and GST was observed compared to the young controls, which was consistent with the increased oxidative stress associated with aging. Furthermore, the results demonstrated that oral CBD treatment was able to restore the enzyme activity levels characteristic of young rats. These findings indicate that CBD may compensate for the loss of antioxidant enzyme expression, as previously shown in other studies [34], and therefore reduce ROS levels, protecting the cells. GR and GPx are enzymes responsible for the reduction and oxidation of GSH, an essential non-enzymatic antioxidant, so their expression is important in protecting against oxidative damage. Similarly, restoring the GST expression also protects the cells from oxidative damage, as this highly expressed liver enzyme conjugates toxic compounds with glutathione to make them less harmful and more easily excretable. However, it is worth noting that, in these studies, changes detected in expression do not always translate into changes in the concentration or activity of these proteins [34,35].

On the other hand, it is confirmed that old rats experience increased oxidative damage to lipids, as evidenced by elevated hepatic levels of MDA, a byproduct of lipid peroxidation, and increased hepatic expression of HO-1d, which is an oxidative stress-inducible protein. Here, oral CBD treatment reduced the high levels associated with aging to the levels observed in young rats, indicating reduced lipid peroxidation and oxidative damage. It is worth noting that previous studies on CBD have shown its ability to increase HO-1d expression, which is contrary to the findings in our study [36]. However, the study was conducted at the cellular level and demonstrated that the effect was dose-dependent, which may explain the difference in results.

The results of this study also showed that the hepatic expression of mRNA of all measured inflammatory mediators, NFκB-p65, NFκB-p52, NFκB-p50, IL-1β and TNF-α, increased in the old group compared to the young group, confirming the presence of an age-related inflammatory response in addition to oxidative stress. The treatment of old rats with CBD allowed for the restoration of expression levels similar to those observed in young rats. These results are consistent, as the expression of the transcription factor NFκB, which can be activated by oxidative stress, leads to the expression of IL-1β and TNF-α which can promote tissue fibrosis [37]. However, to ensure the activation of the transcription factor, the nuclear and cytosolic fractions should have been measured.

In terms of the apoptosis mediators, AIF, BAX, and CASP-1 also showed an increased expression with aging in hepatic tissue, which was reversed with oral CBD treatment. When AIF was released from the mitochondria to the cytosol, it activates apoptosis independently of caspases. Therefore, CBD, by reducing AIF expression, may protect the tissue from the high degree of apoptosis associated with aging. The increase observed in the gene expression of AIF was confirmed when the protein expression was analysed. However, in this case, at the hepatic level, the treatment did not significantly reduce this rise. As mentioned before, this may be due to changes limited to transcription levels and not translation.

In our study, we also investigated Bcl-2 as an anti-apoptotic marker but no significant alterations were observed. Previous studies have found that the protein concentration of anti-apoptotic enzymes such as Bcl-2 and Bcl-XL does not always decrease during apoptosis initiation and that, in some cases, the ratio between pro-apoptotic and anti-apoptotic enzymes seems to be more indicative in cell death, where an increase in proteins like BAX can occur without changes in Bcl-2 or Bcl-XL levels [38]. In accordance with it, in our study, a disbalance in that ration was evident, suggesting that the aging process is associated with an increased apoptosis activation and that CBD treatment could be beneficial helping reducing it.

In our animal model, no significant alteration of the hepatic tissue morphology was observed. We suppose that, since in our experimental model, animals are 13 months old, at that age, a significant alteration of the hepatic tissue is not present yet. However, previous studies observed that, even when morphology is conserved, there could be alterations of the immune system like macrophages [39] or activated neutrophil [40] infiltration. Since the latest was also observed in middle-aged rats, we measured MPO levels in hepatic samples as an indirect measurement of the presence of activated neutrophils. We observed that, even if no significant alteration was evident in the hepatic tissue, a significant increase in MPO was observed in old animals but not in old animals treated with CBD. These results seem to confirm that oxidant, inflammatory, apoptotic, and immune alterations can be seen in advance, when no morphological alteration nor clinical sign is evident yet.

In addition to studying the protective effect of CBD on the liver, which is an important organ involved in detoxification and inflammatory responses, the effect on lung aging was also investigated. The lungs are highly exposed to exogenous free radicals, and continuous exposure to pollution increases the inflammatory response in this tissue [41]. In lung tissue, the same markers with general implications in aging as in liver tissue were studied. In the lungs, an increase in lipid peroxidation with aging (evidenced by increased MDA) was also observed, and it was reversed with CBD treatment, supporting the existence of increased oxidative stress with age for which CBD may have a protective action. The results for the HO-1d gene expression at the pulmonary level did not show any significant difference. Other studies in mouse lung tissue did show an increase in HO-1d mRNA and protein levels [42]. The lack of significance in our results may be due to the lung tissue being less affected by the aging process in 15-month-old rats. However, the high standard deviation that was observed in the old group might affect our results, which could be significantly different to increasing the number of replicates, since a clear tendency was observed. The same can be said for the expression of antioxidant enzymes GST and GPx, where the mean values decreased with aging but not significantly. In the case of GR, CBD increased its expression in old animals, but the values obtained for the young group were unexpectedly low. Overall, the expression of these enzymes in lung tissue is less studied than in liver tissue, and there seems to be less variation in their activity with aging [43]. However, changes in the expression of these enzymes were expected, as previous studies have shown a loss of GR and GPx activity with age in murine models [41], although this may not necessarily be due to changes in expression.

The expression of the inflammation mediators NFκB-p65, TNF-α and IL-1β in the lung also showed an increase in old animals, confirming the age-associated inflammatory response. The production of TNF-α stimulates, among other effects, thrombus formation and apoptosis, and IL-1β production has similar effects [42]. In all three cases, the expression in old rats was reduced to the level of young animals when the CBD treatment was administered, indicating that CBD was also able to protect against age-related inflammation in the lungs. NFκB-p50 and NFκB-p65 dimerise and translocate to the nucleus to act together, so an increase in NFκB-p50 expression is also expected with aging. In our study, NFκB-p50 showed a tendency of an increase in the old group and a reduction when CBD treatment was administered, but the differences were not significant. On the other hand, NFκB-p52 did not show this increase with aging. It is difficult to know whether the NFκB pathway was less altered in the lung than the liver tissue in the early aging process or if these differences were due to other reasons since the activity of NFκB is highly regulated at multiple levels. Further research should investigate the activation and translocation to the nucleus of these proteins.

Finally, the apoptosis markers AIF and CASP-1 showed an increase in expression with aging in lung tissue, which was once again reduced with CBD treatment, suggesting that CBD may decrease the high degree of apoptosis associated with aging. Conversely, the results for BAX expression in the lung did not show significant results. This, along with the lack of significance in other lung results, indicates that the lung tissue was less affected by aging as compared to the liver. In terms of AIF protein levels, similar results to those observed in the gene expression were observed. Our results are consistent with similar ones observed in previous studies [42]. Like the liver, no significant changes were observed in Bcl-2 levels, which, as previously mentioned, highlights the importance of analysing the ratio between pro- and anti-apoptotic proteins.

Overall, it is important to note that not all organs age at the same rate, and CBD does not act in the same way in every organ. In fact, the potential protective role of CBD not only depends on the rate at which an organ ages but also on the presence and proportion of receptors in the corresponding tissue. In this regard, our results were mainly statistically significant in the liver and not in the lungs, suggesting that the lungs were less affected by age-related damage compared to the liver and/or that the CBD could act in a different way in this tissue due to the different receptor distribution.

### Limitations

Our study presents some limitations. In general, the main limitation of this study was the use of 15-month-old rats. At this age, rats can be considered senescent [44], but as we have mentioned, aging is a progressive process in which changes become more evident over time. Therefore, it would be interesting to study the effects of chronic CBD treatment in an experimental model that considers a more advanced age.

On the other hand, for technical and financial reasons, this study does not include some determinations such as RNAseq, phosphoprotein arrays and immunohistochemistry. These data would provide important information to understand patterns of the pharmacological responses of CBD as well as its action mechanism.

Also, this study lacks data from lung histology. Although authors consider that further studies should investigate this more in detail, they also speculate that, as for the liver, no significant alteration should be seen in the pulmonary morphology, especially because our results suggest that lung seemed to be less affected than the liver.

Finally, only MDA was determined to investigate lipid peroxidation. Authors consider that further studies investigating other markers such as 4-HNE would be helpful to confirm our results.

To further investigate the inflammatory cellular state associated with aging and better understand the signalling pathways involved in it, it would be interesting to measure the components of the inflammasome, given its potential relevance to aging [45]. Furthermore, an interesting future direction would be to focus on the molecular mechanisms and signalling cascades through which CBD exerts its effects, as this is essential for the potential application of CBD beneficial effects in medicine.

## 5. Conclusions

This study’s results suggest that chronic treatment with CBD in 15-month-old rats could have beneficial effects in the lung and more significantly in the liver by reducing the levels of inflammatory, oxidative, and apoptotic mediators, and hence the cell damage associated with these three processes inherent to aging.

## Figures and Tables

**Table 1:** Primer sequences for quantitative real-time PCR.

Target Gene		Sequence
*GST*	Forward	TTGAGGCACCTGGGTCGCTCTTTAG
	Reverse	GGTTCTGGGACAGCAGGGTCTCAAA
*GPx*	Forward	CAGTTCGGACATCAGGAGAAT
	Reverse	AGAGCGGGTGAGCCTTCT
*GR*	Forward	GGGCAAAGAAGATTCCAGGTT
	Reverse	GGACGGCTTCATCTTCAGTGA
*HO-1d*	Forward	CTCTTCCAGGGCCGTATAGA
	Reverse	GTCAGGTGTCCAGGGAAGG
*TNF-α*	Forward	ATGAGAAGTTCCCAAATGGC
	Reverse	CTCCACTTGGTGGTTTGCTA
*IL-1β*	Forward	TGTGATGAAAGACGGCACAC
	Reverse	CTTCTTCTTTGGGTATTGTTTGG
*NFκB-p65*	Forward	CGAGCTCTAAAGAGTCCCAAG
	Reverse	CCTCTGGGCCAATCAAATC
*NFκB-p52*	Forward	TGGAACAGCCCAAACAGC
	Reverse	CACCTGGCAAACCTCCAT
*NFκB-p50*	Forward	CACCTCTTCTCAAAGCAGCA
	Reverse	TCCAGGTCATAGAGAGGCTCA
*CASP-1*	Forward	GCCTGTTCCTGTGATGTGGAG
	Reverse	TGCCCACAGACATTCATACAGTTTC
*AIF*	Forward	AGTCGTTATTGTGGGGTTATCAAC
	Reverse	TTGGTCTTATTTAATAGTCTTGTAGGC
*BAX*	Forward	GTGAGCGGCTGCTTGTCT
	Reverse	GTCCCGAAGTAGGAGAGGA
*18S*	Forward	TCCGATAACGAACGAGAC
	Reverse	CTAAGGGCATCACAGACC

**Table 2:** Primary antibodies used for the Western blotting technique.

Antibody	Type	kDa	Dilution	Catalogue N°	Producer
NFκB-p65	RbP	65	1:1000	14-6731	eBioscience
NFκB-p52	RbP	52–100	1:200	14-6733	eBioscience
TNF-α	RbP	150	1:4000	500-P72	PeproTech
AIF	RbP	67	1:1000	5318	Cell signaling
Bcl-2	RbP	26	1:1000	2870	Cell sgnaling

## Data Availability

The data presented in this study are available on request from the corresponding author upon reasonable request.

## Abbreviations

8-OH-dG	8-hydroxy-2′-deoxyguanosine
AIF	Apoptosis-inducing factor
BAK	Bcl-2 antagonist/killer
BAX	Bcl-2-associated X protein
Bcl-2	B-cell lymphoma 2
Bcl-xL	B-cell lymphoma-extra large
BHT	Butylated hydroxytoluene
CBD	Cannabidiol
COPD	Chronic obstructive pulmonary disease
GPx	Glutathione peroxidase
GR	Glutathione reductase
GSH	Glutathione
GSSG	Glutathione disulfide
GST	Glutathione S-transferase
HO	Nuclear heme oxygenase
HO-1d	Nuclear heme oxygenase-1
IL-1β	Interleukin 1 beta
MDA	Malondialdehyde
MPO	Myeloperoxidase
MOMP	Mitochondrial outer membrane permeabilisation
NFκB	NADPH oxidase
PUFAs	Polyunsaturated fatty acids
ROS	Reactive oxygen species
TBA	Thiobarbituric acid
THC	Tetrahydrocannabinol
TNF-α	Tumour necrosis factor alpha
